# Satisfying Product Features of a Fall Prevention Smartphone App and Potential Users’ Willingness to Pay: Web-Based Survey Among Older Adults

**DOI:** 10.2196/mhealth.9467

**Published:** 2018-03-27

**Authors:** Peter Rasche, Alexander Mertens, Christopher Brandl, Shan Liu, Benjamin Buecking, Christopher Bliemel, Klemens Horst, Christian David Weber, Philipp Lichte, Matthias Knobe

**Affiliations:** ^1^ Institute of Industrial Engineering and Ergonomics Department of Mechanical Engineering RWTH Aachen University Aachen Germany; ^2^ Department of Emergency Medicine Massachusetts General Hospital Boston, MA United States; ^3^ Hand and Reconstructive Surgery Department of Trauma University Hospital of Giessen and Marburg Marburg Germany; ^4^ Department of Orthopaedic Trauma University of Aachen Medical Center RWTH Aachen University Aachen Germany

**Keywords:** prevention, cell phone, accidents

## Abstract

**Background:**

Prohibiting falls and fall-related injuries is a major challenge for health care systems worldwide, as a substantial proportion of falls occur in older adults who are previously known to be either frail or at high risk for falls. Hence, preventive measures are needed to educate and minimize the risk for falls rather than just minimize older adults’ fall risk. Health apps have the potential to address this problem, as they enable users to self-assess their individual fall risk.

**Objective:**

The objective of this study was to identify product features of a fall prevention smartphone app, which increase or decrease users’ satisfaction. In addition, willingness to pay (WTP) was assessed to explore how much revenue such an app could generate.

**Methods:**

A total of 96 participants completed an open self-selected Web-based survey. Participants answered various questions regarding health status, subjective and objective fall risk, and technical readiness. Seventeen predefined product features of a fall prevention smartphone app were evaluated twice: first, according to a functional (product feature is implemented in the app), and subsequently by a dysfunctional (product feature is not implemented in the app) question. On the basis of the combination of answers from these 2 questions, the product feature was assigned to a certain category (must-be, attractive, one-dimensional, indifferent, or questionable product feature). This method is widely used in user-oriented product development and captures users’ expectations of a product and how their satisfaction is influenced by the availability of individual product features.

**Results:**

Five product features were identified to increase users’ acceptance, including (1) a checklist of typical tripping hazards, (2) an emergency guideline in case of a fall, (3) description of exercises and integrated workout plans that decrease the risk of falling, (4) inclusion of a continuous workout program, and (5) cost coverage by health insurer. Participants’ WTP was assessed after all 17 product features were rated and revealed a median monthly payment WTP rate of €5.00 (interquartile range 10.00).

**Conclusions:**

The results show various motivating product features that should be incorporated into a fall prevention smartphone app. Results reveal aspects that fall prevention and intervention designers should keep in mind to encourage individuals to start joining their program and facilitate long-term user engagement, resulting in a greater interest in fall risk prevention.

## Introduction

### Background

Falls and fall-related injuries pose a major threat to older adults’ health and are associated with increased morbidity and mortality [1-3]. Indication from the literature [4,5] suggests that older adults tend not to be sufficiently aware of their potential for falls or their fall risk. In addition, the literature [6-9] suggests that client outcomes vary with the type of treatment prescribed, the equipment of the clinic, and the health professional’s abilities. Hence, preventive measures are important for fall prevention and reduction of associated injuries over time.

One method is enabling older adults to self-assess their possible fall risk and thereby enable them to become aware of their potential fall risk [4,10,11]. A promising attempt is to incorporate health apps in this context, given that the use of health apps is rising among older adults [12-18]. A health app is an unobtrusive way to offer potential support in terms of prevention activities [19-21]. The sensor technology built into smartphones is precise enough to allow extensive data collection to record the user's state of health [14,22,23]. Different research projects have already addressed the topic of fall prevention using varied approaches [14,24]. The FARSEEING project, funded by the European Union (EU), developed a smartphone app to measure users’ fall risk based on daily activities as it incorporated an adapted version of the Timed-Up and Go test [14,25]. Users of this product were able to get real-time feedback regarding their individual fall risk. An intervention or decision about treatment was not included in this app. The question on how to decide about the right treatment was investigated by the ProFouND project, also funded by the EU [26]. Within this project, an app for health care professionals was developed, which can help the decision process regarding a certain patient [27]. A different approach was undertaken by the FallCheck project at Coventry University [28]. They developed an app that helps older adults identify typical tripping hazards within their home [24]. The question on how to motivate and instruct physical exercises for older adults in their home was investigated by the iStoppFalls project [29]. This project showed that a continuous exercise plan could decrease older adults’ fall risk. Within this project, older adults performed physical exercises on their own. They were instructed and motivated by exergames on a television. The correct performance of the exercises was supervised using information and communication technology such as activity tracker [29].

However, there has been no app that combines these approaches into a single product. To design such a fall app, it is necessary to determine potential users’ expectations regarding such a fall prevention smartphone app.

### Aim of This Study

This study investigates which product features potential users expect of a fall prevention app and how these features would contribute to users’ satisfaction with such an app. A Web-based survey was performed questioning older adults who were not participants in a prior fall prevention–related study conducted by the authors [21,30].

### Research Questions

In summary, the main research questions of this study were as follows:

1. Which product features should a fall prevention app have, to increase the likelihood of acceptance by the elderly and consequently reduce the risk of falls in the elderly population?
2. Which product features increase or decrease use of such an app?

This study aims to provide guidance on how to design a user-friendly and acceptable fall prevention app for older adults.

## Methods

### Design

An open, self-selected, Web-based survey was designed to investigate the research questions. The survey was designed in German and provided for German-speaking Internet users. A Web-based survey was used, given it is a suitable method to reach individuals with particular characteristics or interests in a short period without any limitations on physical space [31,32].

On the basis of research questions, the aim of this survey was to collect data regarding expected product features of a fall prevention app. Expectation was measured using the Kano technique [33]. This is a preference classification technique to identify user requirement and expectation during the early product development stage [34]. Within the health care sector, this technique is not a well studied and less used approach but potentially a suitable one to design health care interventions and services according to users’ needs [35-37].

#### Investigated Product Features of a Fall Prevention App

For this study, features were identified based on literature of former fall prevention projects and expert interviews. These product features are related to 6 different topics, including detection of a fall risk, decision making about a treatment or intervention, comfort functions, advice and support functions, physical exercise advice, and cost coverage by health insurance companies (refer to Table 1).

##### Detection

Related to the topic detection, 2 product features were identified to be relevant: (1) the automatic detection of the risk of falling through the app during general everyday activities and (2) the detection during the execution of standardized tests. Both product features have already been implemented in the “FARSEEING” project [14,25]. However, it remains to be seen whether potential users prefer continuous data collection in everyday life, based on which a fall risk is determined or whether they prefer to carry out explicit test procedures for detection.

**Table 1:** Investigated product features of a fall prevention app. C: criteria.

Topic		Description
**Detection**		
	C1	The app recognizes your fall risk based on a standardized test.
	C2	The app automatically detects your risk of falling if you carry your smartphone with you.
**Decision making**		
	C3	The app leaves the decision to treat your fall risk to your health care professional.
	C4	The app itself decides about the treatment of your fall risk.
**Comfort**		
	C5	In addition to the risk of falling, other health data such as medication can be stored in the app.
	C6	You can share the results of your fall evaluation with your health care professional or friends and family by email.
	C7	The following treatment appointments can be stored in the app.
**Advice and support**		
	C8	The app contains a checklist of typical tripping hazards.
	C9	The app contains a guideline on how to react in the case of a fall for the falling person and the person who is helping.
**Physical exercise**		
	C10	The app includes physical exercises to reduce your risk of falling.
	C11	The app includes an ongoing workout program to reduce the risk of falling.
	C12	The training integrated into the app is supervised by a therapist.
	C13	The training integrated in the app can be adapted to your personal needs (scope of training, exercises, time expenditure, and so on).
	C14	Within the integrated training, individual goals can be defined.
	C15	New social contacts can be made while using the app.
	C16	The training within the app includes playful elements such as awards, rankings, and so on.
**Cost coverage**		
	C17	The costs of the app are covered by the health insurance company.

##### Decision Making

After the detection of a certain fall risk, the second question is how to manage this risk and which interventions or treatments could be applied [26,27]. Two potential product features were included in the study: (1) a health professional decides on the possible treatment measures based on data collected by the app and (2) the app itself makes a recommendation for the treatment of a possible fall risk. Here too, the question arose as to what would be preferred by potential users.

##### Comfort

Several product features related to certain comfort of an app were derived from literature. Product features include additional data storage, sharing data, and setting reminder for medical appointments. Mendiola et al identified these features to be valuable features of health apps [19]. Other health apps included such functions to increase users’ adherence to the app [38,39].

##### Advice and Support

Another question within this study was whether potential users would appreciate a checklist of typical tripping hazards as what the FallCHeck website offers [24]. Furthermore, the option of an emergency guideline to guide users’ actions after a fall incident was included in this investigation [38].

##### Physical Exercise

Physical exercise is known to reduce a potential fall risk [4,40-43]. Hence, the integration of physical exercises in the fall prevention app seemed to be an important aspect. In this context, 7 different product features were investigated. First, including physical exercises itself was questioned. Second, participants were asked whether they prefer a continuous exercise plan or not. Third, participants were asked whether they want to have a therapist to oversee their training such as what the Otago program includes [42,43]. It was further questioned whether the training should be adaptable to personal needs as, for example, types of exercise or time spent on training. Whether potential users want to set individual training goals was asked as fifth product feature. This was included as a study by Schlomann et al implicates older adults to exceed their abilities in physical training by missing individual training goals and exercises [44]. Lastly, 2 product features, one regarding making new social contacts and another regarding gamification were included in this investigation as both features were recommended by Mendiola et al to be valued ones in health apps [19].

##### Cost Coverage

The last product feature investigated was cost coverage by a health insurance company. With this characteristic, it should be examined whether the general customary assumption of costs by the health insurance company is desired or presupposed in the case of a fall prevention app [45].

#### Kano Technique

Each of the 17 predefined product features was evaluated twice: first, according to a functional (product feature is implemented in the app), and subsequently by a dysfunctional (product feature is not implemented in the app) question. This technique is based on the Kano model, widely used in the user-oriented product development realm [35-37].

Both types of questions were asked in succession. Five possible answers were available for both questions:

- I would be very happy
- I take that for granted
- I don’t care
- I barely accept this
- That would annoy me

Through the combination of answers of functional and dysfunctional questions, the classification of a product feature was derived, as defined earlier in the section [29]. This technique differentiates 7 categories.

- Must-be (M): These product features are taken for granted when fulfilled but result in dissatisfaction if they are not fulfilled.
- One-dimensional (O): These product features result in satisfaction when fulfilled and dissatisfaction when not fulfilled. These are product features that are spoken and the ones in which companies compete.
- Attractive (A): These product features provide satisfaction when achieved fully but do not cause dissatisfaction when not fulfilled. These product features are not expected by a normal customer and thereby have the potential to please the customer.
- Indifferent (I): These product features refer to aspects that are neither good nor bad, and they do not result in either customer satisfaction or customer dissatisfaction.
- Reverse (R): These product features refer to a high degree of achievement, resulting in dissatisfaction and to the fact that not all customers are alike. For example, some customers prefer high-tech products, whereas others prefer the basic model of a product and will be dissatisfied if a product has too many extra features.
- Questionable (Q): Product features in this category should be reviewed. It is most likely that the questions for this product feature were not appropriate for the app of the Kano technique.

Generally, a product feature is assigned to the category most frequently rated [33]. To verify the results of the encoding by the Kano technique, 2 different decision rules are available: (1) category and total strength [46] and (2) the Fong test, if category and total strength led to no clear categorization [47].

Furthermore, customer satisfaction (CS) coefficients were calculated for all investigated products. This coefficient is a measure of whether a product feature can explicitly increase the satisfaction of the potential user or whether the existing product characteristic can only prevent the potential user from being dissatisfied with the overall product [48,49]. According to this definition, the CS coefficient is divided into 2 components. One component has a positive sign and describes whether the satisfaction of the potential user can be increased beyond an expected level by fulfilling the product characteristic (CS+). The second component of the CS coefficient has a negative sign and thus indicates to what extent the satisfaction of the potential user would fall below an expected level if this product characteristic is not taken into account in the overall product (CS−). If the individual component of the CS coefficient (CS+ or CS−) has an absolute value greater than .5, this component and thus the CS coefficient of the associated product characteristic is assumed to be significant [48,49].

#### Willingness to Pay for a Fall Prevention App

Willingness to pay (WTP) was assessed as monthly payment [50,51]. A potential fall prevention app should be available in the common app stores for users to explore use without having to purchase it (Freemium Business model) [13,50]. Hence, necessary revenues to develop and maintain the app need to be generated afterward, meaning monthly payments by a subscription model or in-app purchases. WTP was studied to explore how much money potential users would spend on in-app purchases so that developers could estimate potential revenues [50]. This topic was addressed after participants rated the 17 product features. Participants were able to enter an amount between 0 and several hundred euros, including 2 decimals.

#### Characterizing Participants of This Survey

##### Measuring Health Status

Participants self-reported known medical conditions and chronic diseases. Health competency of participants was measured using an adapted version of the European Health Literacy Scale with 16 items [52]. Corresponding statements were evaluated on a 4-point Likert scale (1=not correct and 4=fully correct). Subsequently, final score was calculated according to Röthlin et al [53]. Final score ranges between 0 points and 16 points, with a high score indicating a high health competency [52].

Quality of life was assessed with the EuroQol Questionnaire (EQ5D-3L) [54], which is a validated tool for measuring general health-related quality of life. It consists of 5 items (mobility, self-care, usual activities, pain or discomfort, and anxiety or depression), each of which is rated as causing “no problems,” “some problems,” or “extreme problems.” The EQ5D-3L thus distinguishes 243 unique health states. Each unique health state has a utility score which lies within a range between 0 (poor health) and 1 (perfect health). This single EQ5D-3L summary index score was used in this study [54].

##### Measuring Fall Risk

Given that purpose of this study was to investigate the desired functions of a fall prevention app, measurements to access participants’ objective and subjective risk of falling were included as measured by the individual’s history of falls in the past year [55], the Aachen Falls Prevention Scale (AFPS) [10] and the short Falls Efficacy Scale-International (FES-I) [56].

Objective fall risk was determined retrospectively based on the individual’s history of falls in the past year [55]. Using fall risk screening criteria, participants reporting ≥2 noninjury falls in the past year or ≥1 injury fall were categorized as “fallers”; participants reporting no falls were categorized as “nonfallers”; the remaining subjects were defined as indifferent [55,57]. On the basis of the answer whether participants have fallen or not, detailed questions about the falls and their circumstances were asked.

Subjective fall risk was accessed by 2 aspects: the AFPS and the FES-I. The AFPS is a self-assessment test containing 3 steps participants had to perform in this survey [10]. First, participants answered a self-test containing 10 standardized yes or no questions (positive criterion≥5 “Yes”). Questions addressed relevant risk factors derived from several fall risk assessment tools [10]. Second, participants performed a balance test on their own. During this test, participants had to position their feet next to each other and hold this position for at least 10 s without compensatory movement (positive criterion: compensatory movement). In the third and final step, participants rated their “subjective risk of falling” on a 10-point Likert scale based on the results of the first 2 steps. A score of more than 5 points on this scale indicates a certain fall risk (cutoff score >5 points).

The short FES-I questionnaire was used to assess participants’ Fear of Falling (FOF) [56,58] to investigate whether certain product features are related to this psychological aspect of patients’ fall risk. This questionnaire contains 7 items rated on a 4-point Likert scale (1=not at all concerned to 4=very concerned). The results of all 7 items are added into a final score, ranging from 7 (no concern about falling) to 28 (severe concern about falling) [56].

##### Measuring Technology Readiness

Technology readiness was included as it might influence the use of modern information and communication technology as well as the engagement with these products [59]. It is calculated based on 12 standardized items which are rated on a 5-point Likert scale (1=not correct and 5=fully correct). For negatively formulated items, the scale is converted so that a high point value corresponds to high technology readiness. Subsequently, final score is calculated by mean value over all 12 items; thus, the score ranges between 1 and 5 points [59].

##### Measuring Attitude Toward a Fall-Related Intervention

Participants’ attitude toward the fictive fall prevention app was accessed using the Attitudes Falls Related Intervention Scale (AFRIS) [60,61]. Hence, it was possible to evaluate whether a participant is generally interested in a fall intervention program or not. The questionnaire consists of 6 items rated on a 7-point Likert scale (1=I totally disagree to 7=I totally agree). The results of all 6 items are summed up to a final score, ranging from 6 points (no intention) to 42points (absolute intention) [60,61].

### Questionnaire

The questionnaire started by presenting a short description of the context, followed by demographic questions regarding participants’ age, educational level, and health status. Next, the participants performed a self-assessment of their subjective fall risk and their FOF. In addition, participants fulfilled a standardized questionnaire regarding their technological readiness. Then participants evaluated the 17 product features regarding described measurements and entered an amount of money they would spend to use such an app. Finally, participants fulfilled the AFRIS questionnaire measuring whether participant would engage with a prevention program or not.

### Data Collection

Data were collected between September 1 and October 31, 2017. The questionnaire was programmed and made available on a website hosted using the Unipark software (QuestBack GmbH, Cologne, Germany) [62]. The survey was introduced as a study examining the desired functions of a fall prevention smartphone app (see Multimedia Appendix 1).

All participants were informed about the duration of the survey, data storage, and the leading investigator. Each participant decided to take part in this survey voluntarily by following the designated link to the survey. A monetary incentive of €3 per participant was offered for participation.

The survey was tested properly by 2 independent examiners with regard to wording and technical functionality. The survey included 63 items, distributed over 16 different pages. Participants were able to review their entries per page before moving on.

### Recruitment

Different channels of recruitment were applied to reach a broad range of potential participants in this open survey. It was avoided to address existing users of the Aachen Fall Prevention App or participants of a different fall prevention−related study of the authors as these participants might have a different opinion about the design and necessity of features of a fall prevention smartphone app [21,30]. Further exclusion criteria or screening questionnaires were not applied. The sampling procedure was nonprobabilistic, and respondents were selected based on their voluntary willingness to participate [31].

The Web-based survey was promoted by a Clickworker advertisement, targeting persons aged older than 60 years [63]. This method of recruitment was chosen because this platform offers the possibility of providing monetary incentives. Finally, the link to the open Web-based survey was distributed in a mailing list for elderly who are regularly taking part in studies at the Institute of Industrial Engineering and Ergonomics of RWTH Aachen University, Germany. In all cases, the recruitment was based on the same text as shown in Multimedia Appendix 1.

In total, 157 unique individuals visited the website of the Web-based survey. The identification of different individuals was performed using the Unipark software based on Internet Protocol address and cookie function. Of 157, 49 visitors never started the survey. Nine discontinued completing the survey. In total, 99 visitors finally participated in the survey and completed the whole questionnaire. Three of these were excluded for analysis as attention checkmarks within the questionnaire showed inappropriate data quality. The participation rate was thus 68.8% (108/157), and the completion rate was 63.1% (99/157). The average duration of completing the survey was 17 min and 12 s, with a median of 15 min and 13 s.

### Statistical Analysis

Data were analyzed with SPSS 22 (IBM, USA) and MatlabR2017b (The MathWorks, USA). Investigated product features were assigned to the corresponding category according to the Kano technique. Furthermore, the category strength and total strength of each product feature are provided. In case that applying category and total strength rule resulted in an indifferent categorization, Fong test was performed . In addition, CS coefficients were calculated to analyze and prioritize investigated product features in terms of their contribution to users’ satisfaction with a fall prevention app.

### Ethics Statement

The Ethics Committee at RWTH Aachen Faculty of Medicine authorized this study and its ethical and legal implications in its statement EK236/16.

## Results

### Participants

In total, 96 participants took part in this study. The mean age was 63.8 years (SD 7.02), and 51% (49/96) were female. All participants lived autonomously in a flat or house. In all, 29% (19/96) lived together with their family, 56% (54/96) with their marriage partner or companion, and 29% (28/96) lived alone. The level of education varied from minor educational degree to postsecondary degree.

About 78% (75/96) of all participants stated to use a smartphone; however, none of the participants had any experience at all with a smartphone app aiming to prevent falls, and 19% (19/96) stated to already use health apps.

#### Health Status

About 64% (62/96) of all participants suffered from a chronic disease such as high blood pressure (37%, 36/96), back pain (20%, 20/96), cardiovascular disease (16%, 16/96), or diabetes (12%, 12/96).

Health literacy varied a median score of 15.00 points (interquartile range, IQR 4) on a range from 0 to 16 points, indicating a high qualification and interest in managing personal health.

Median score of quality of life as measured by the EQ5D-3L was 0.716 (IQR 0.365) ranging from 0 to 1 and thereby indicating a good quality of life within the sample.

#### Fall Risk

Fifty-eight (60%, 58/96) participants stated that they had fallen within the last year. Furthermore, 31 (32%, 31/96) reported to have fallen at least once within the last year, and finally, 7 participants (7%, 7/96) indicated to have fallen between 2 and 3 times within the last year. Seven of these 38 participants, who had fallen, needed to visit the hospital for medical care as a direct result of their fall. Hence, 84 participants (87%, 84/96) were classified as “nonfallers,” and 12 participants (12%, 12/96) were classified as “fallers.” Fallers are defined as participants reporting ≥2 noninjury falls in the past year or ≥1 injury fall. Main reasons for falling were tripping (26%, 25/96), dizziness (4%, 4/96), and physical weakness (4%, 4/96), whereas combination of reasons are possible as multiple answers were allowed.

For 8 participants (8%, 8/96), the self-test (step 1 of the AFPS: 10 standardized questions, positive criterion ≥5 points) was positive. In contrast, 6 (6%, 6/96) participants did not pass the balance test (step 2 of the AFPS: balance test, positive criterion: compensatory movement). After steps 1 and 2 of the AFPS had been completed, 88 (91%, 88/96) participants estimated their “subjective risk of falling” to be low (≤5 points), and 8 (8%, 8/96) participants rated their “subjective risk of falling” as high (>5 points). The overall median value was 2.0 points (IQR 2.0) on a 10-point Likert scale ranging from 0 to 10 points.

The median FOF was 8.0 points (IQR 2.5) on a scale ranging from 7 to 28 points, suggesting a low FOF.

#### Technology Readiness

The median technology readiness was 4.0 points (IQR 0.917) on a scale ranging from 1 to 5 points, indicating a high technology readiness.

#### Attitude Toward a Fall-Related Intervention

Median score for the attitude toward a fall-related intervention was 24.0 points (IQR 9.5) ranging from 6 points (no intention) to 42points (absolute intention), indicating moderate intention to attend a fall intervention program.

### Classified Product Features According to Kano Technique

Table 2 presents the investigated product features assigned to the corresponding category according to the Kano technique. Furthermore, the category strength and total strength of each product feature are provided. The Fong test was performed in case that category and total strength rule did not lead to a clear categorization. According to these rules, all product features were valid categorized.

**Figure 1:**
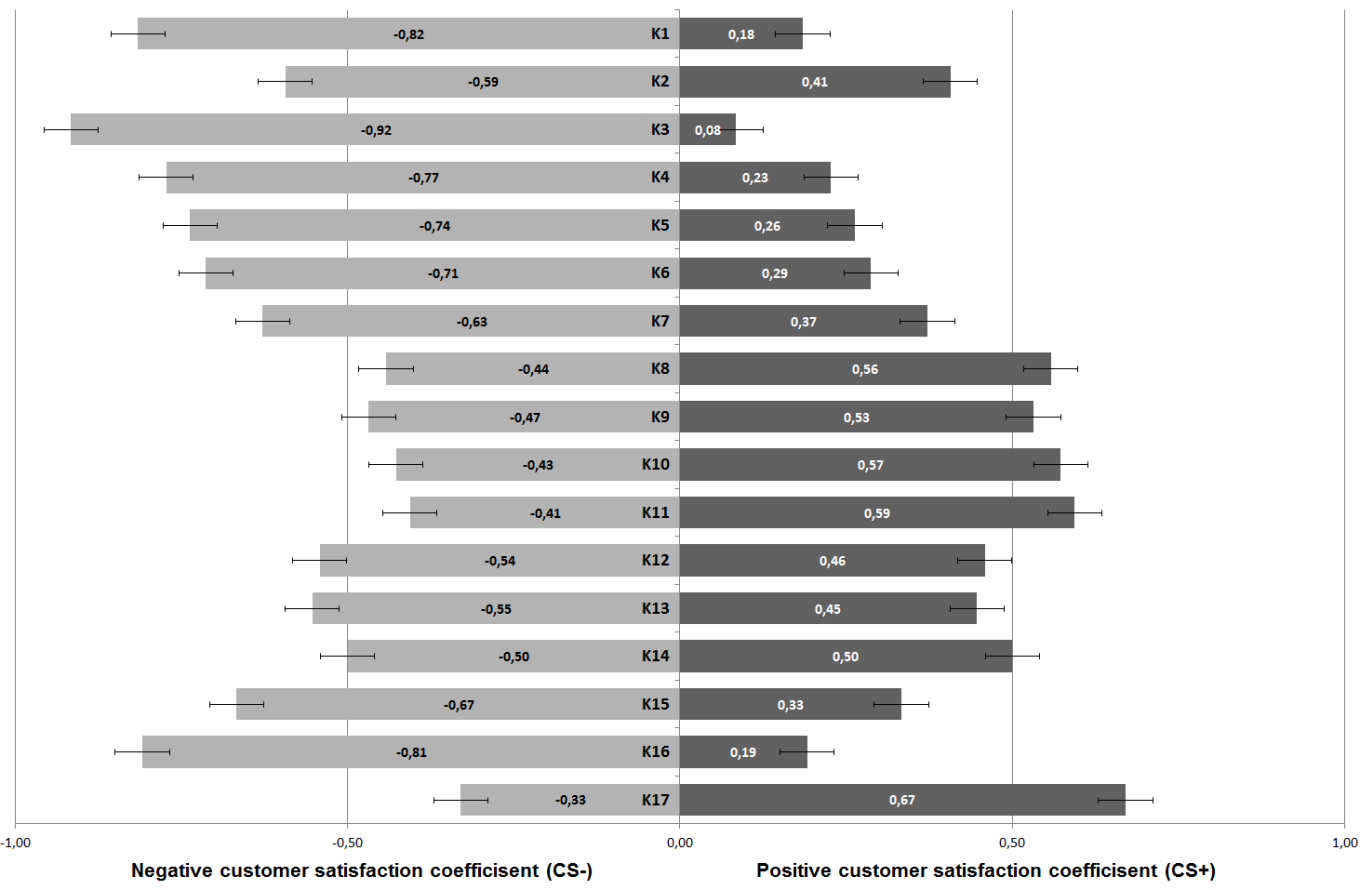
Customer satisfaction (CS) coefficients of investigated product features.

Figure 1 provides CS coefficients for each product feature. Both components of the CS coefficient (CS+) and (CS−) are shown as one bar, whereas darker color indicates (CS+) values.

### Willingness to Pay for a Fall Prevention App

**Figure 2:**
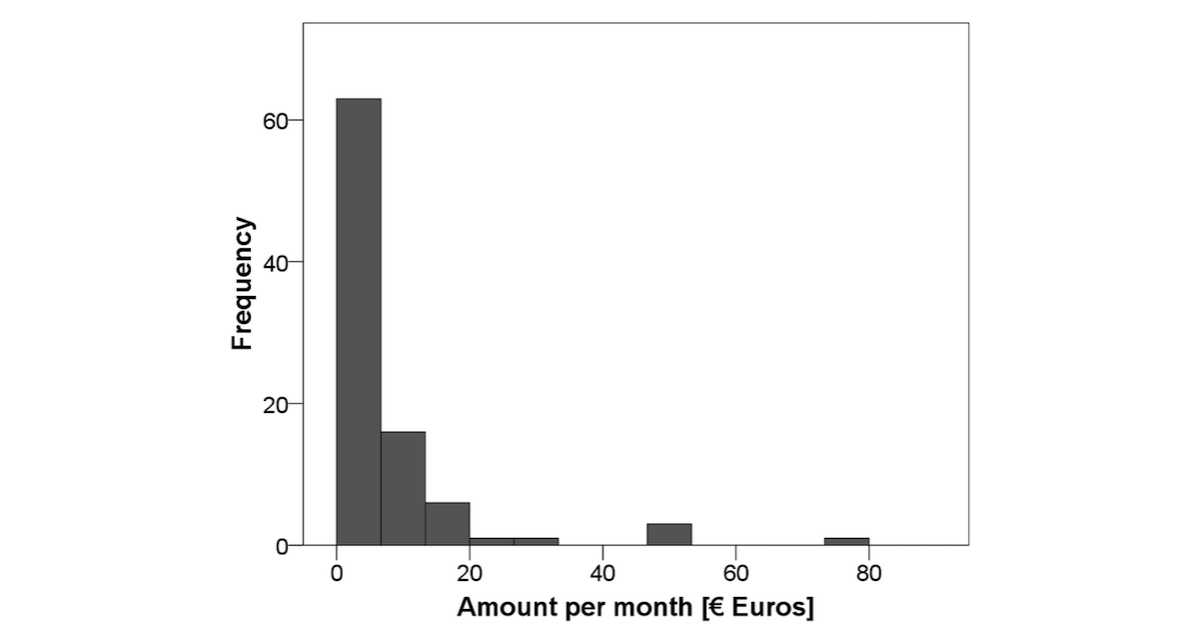
Histogram for the willingness to pay.

One of the last questions asked was how much money participants would spend per month to use a fall prevention smartphone app. Results showed a wide variety ranging from €0 to €80 per month with a median amount to spend €5 per month (IQR 10; see Figure 2).

**Table 2:** Investigates functions of a fall prevention app and their results. C: criteria. N/A: not applicable. Sig: significant categorization according to Fong test.

Topic		Product feature	Category	Category strength (%)	Total strength (%)	Fong test
**Detection**						
	C1	Fall risk identification by standardized test	Must-be	60.42	95.80	N/A
	C2	Automatically identification of fall risk	Must-be	17.71	94.80	
**Decision**						
	C3	Decision about treatment by health care professional	Must-be	82.29	99.00	
	C4	Decision about treatment by app	Must-be	50.00	91.70	
**Comfort**						
	C5	Additional data storage	Must-be	46.88	99.00	
	C6	Data sharing via email	Must-be	41.67	97.90	
	C7	Appointment reminder	Must-be	25.00	97.90	
**Advice and support**						
	C8	Checklist of typical stumbling blocks	Attractive	11.46	99.00	Sig
	C9	Guideline in case of a fall incident	Attractive	6.25	97.90	Sig
**Physical exercise**						
	C10	Description of physical exercises to reduce fall risk	Attractive	14.58	100.00	
	C11	Continuous workout program	Attractive	18.75	100.00	
	C12	Training integrated is supervised by a therapist	Must-be	8.33	100.00	Sig
	C13	Individualization of training within app	Must-be	10.42	97.90	Sig
	C14	Define individual training goals	Must-be	0.00	97.90	Sig
	C15	Make new social contacts	Must-be	33.33	100.00	
	C16	Serious gaming elements	Must-be	60.42	97.90	
**Cost coverage**						
	C17	Cost coverage by health insurer	Attractive	33.33	97.90	

Separate univariate one-way analyses of variance (ANOVA) revealed no significant effects of the between-subject factors “age,” “gender,” “education,” “Health Literacy Scale,” “number of chronic diseases,” “faller or nonfaller,” “FES-I,” “technology readiness,” or “attitude toward a fall-related intervention” for the WTP. Separate univariate ANOVAs revealed significant effects for the WTP if the categorization of the product features is treated as between-subject factor. Regarding 4 product features, significant effects were revealed (decision about treatment by app, *F*_2,68_=3.593, *P*<.05; description of physical exercises to reduce fall risk, *F*_1,68_=8.964, *P*<.05; continuous workout program, *F*_1,68_=4.87, *P*<.05; and make new social contacts, *F*_1,68_=1.124, *P*<.05). Regarding the feature “decision of treatment by app,” 8 participants categorized this feature as questionable. Within these 8% (8/96), mean amount spent per month was higher than within the group of participants who categorized this feature as attractive or must-be one. In case of the other 3 features, participants who rated these as attractive ones were also willing to spend a higher median amount of money to use such an app.

## Discussion

### Principal Findings

In an exploratory approach, requirements were ascertained which may influence users’ acceptance of a fall prevention smartphone app. Seventeen product features were rated according to the Kano technique. According to the calculated category and total strengths as well as the Fong tests, all product features have been validly categorized by the Kano technique. In total, 12 must-be product features were identified ranging from “automated detection of a fall risk,” over “storing additional medical data” within the app up to “letting a health care professional and the app make decisions about the type of intervention treatment.” Five remaining product features were identified as so-called attractive ones. These are features participants do not expect a fall prevention app to have but would be attracted to the app if it would have this function. Product features within this group were mainly related to offering a physical training program via the app, including a personalized workout plan and individual goal setting. In addition, a checklist of typical tripping hazards and an action guide in case a fall occurs were identified as attractive product features.

Detailed analysis using CS coefficient calculations revealed that all except one product feature significantly increased or prevented loss of users’ satisfaction. The exception was the product feature “define individual training goals,” as this feature showed no significant contribution regarding an extent of satisfaction or dissatisfaction.

Missing fall risk detection, missing consultation of a health care professional regarding the treatment, and missing serious gaming aspects within the app were rated highest among negative CS coefficients and therefore would greatly reduce users’ acceptance (CS_decision by health care professional_=−0.92, CS_detection_=−0.82, and CS_serious gaming_=−0.81). Product features, for example, cost coverage by health insurance companies, a continuous workout plan, and instructions for home-based physical exercises, both aiming to decrease a fall risk, would significantly increase users’ acceptance and attraction to a fall prevention smartphone app (CS_cost coverage_=0.67, CS_workout plan_=0.59, and CS_exercise instructions_=0.57).

These results impressively show how difficult it is to design a user-accepted fall prevention app as all suggested product features of a possible app were evaluated as “must-be” or “attractive” product features. Hence, a fall prevention app would need numerous features to be developed and implemented. One reason for the categorization might be that participants had not ever used a fall prevention smartphone app and thereby desired as many features as possible. After their first experience, they might have a more concrete idea of the features they might need. Nevertheless, CS coefficients indicate a clear priority order among investigated must-be and attractive product features. Developers should address the topic of decision making and fall risk detection as well as serious gaming aspects in their potential app. In addition, clear instructions for exercises and workouts that would decrease a fall risk, as well as cost coverage by health insurance companies would increase users’ acceptance as well as their attraction to use such an app.

Investigated WTP revealed a median amount of €5 per month (IQR 10) participants would invest to use a smartphone app, incorporating the 17 products features as they rated them. This amount is similar to the average price of paid apps within the Apple App Store and Google Play as measured in 2017 [64].

Different independent ANOVA indicate that participants who rated the features “description of physical exercises to reduce fall risk,” “continuous workout program,” and “make new social contacts” as attractive ones were willing to pay a higher amount of money to use such an app as participants who did not. Only for the fourth significant product feature (decision about treatment by app) was a reverse correlation identified. Participants who did not rate this feature as an attractive one were willing to pay a higher amount of money. This might be because of the 8% (8/96) of participants who categorized this feature (decision about treatment by app) as questionable. Results show that attractive product features result in a higher WTP. Features such as “description of physical exercises to reduce fall risk,” “continuous workout program,” and “make new social contacts” would motivate potential users to spend significantly more money for the ability to use a fall prevention app as the other investigated ones.

### Comparison With Prior Work

#### Product Features

The FARSEEING demonstrated the technical possibility of measuring users’ fall risk during daily activities by a special device [14,25]. This study could extend this knowledge as it shows that potential users would appreciate an automated fall risk assessment and detection as this would be done by a fall prevention smartphone app. The survey revealed detection of users’ potential fall risk to be a must-be product feature, whereas automated detection without performing standardized test as a Timed-Up and Go test would be preferred (product features: criteria (C)1; C2).

An intervention or decision about treatment was not included in the app of the FARSEEING project. Nevertheless, this was considered to be the next mandatory step to design an innovative fall prevention app. Therefore, the product features “decision of treatment by health care professional” (product feature: C3) and “decision of treatment by app” (product feature: C4) were investigated in this survey. Related results show that the decision about a necessary intervention is a must-be product feature, whereas “decision of treatment by health care professional” even reached the highest negative CS coefficient, indicating that missing this feature would reduce potential users’ satisfaction significantly. Hence, future research should try to include such options into a fall prevention app. A recent attempt was made by the ProFouND project [26]. Within this project, an app for health care professionals was developed, which should support them during the decision process about the treatment of a certain patient [27].

Mendiola et al identified in their content analysis 12 features a health app might profit from [19]. In this study, 3 of these 12 features were investigated regarding their relevance for a fall prevention app. In this survey, participants classified product features as additional data storage, data sharing via email, and appointment reminder as must-be features of a fall prevention app. Hence, this survey supports the content analysis of Mendiola et al by empirical data.

Physical exercise is known to be an important factor to reduce a person’s fall risk [4,29,40-43]. Therefore, it was part of this study to investigate how a physical exercise program should be designed according to potential users’ expectations. The results indicate that participants are aware of the positive effect physical exercise has on a potential fall risk, as all related product features were at least rated as must-be features. Interestingly, a continuous workout program such as the Otago program and the instruction of physical exercises within the app were even classified as attractive features. On the basis of the results from this study, physical exercise interventions within a fall prevention app should be designed as continuous workout programs, which are supervised by a health professional or clinician. Nevertheless, instructions for the exercises themselves should be available within the app; so potential users are able to exercise on their own. Such an exercise program is also expected to support social contact and add an element of fun or be more satisfying as serious gaming elements were classified as must-be features.

These results are similar to the findings of Danbjørg et al [65]. Their study revealed that older adults appreciate a personal therapist as the therapist could motivate them through comments and personal social contact. Furthermore, they did identify “motivation by competition” to be a highly relevant factor to motivate older adults to perform physical activities [65]. The feature of defining individual training goals was rated ambivalent. This result is quite reasonable as a study by Schlomann et al identified diverse acceptance of fixed training goals within fitness apps among older adults [44]. Their study revealed that the elderly try to achieve a socially accepted goal, as it is suggested by a fitness app, even if this is exceeding their physical abilities [44].

#### Willingness to Pay

Participants’ WTP was quite small compared with the expected product features of a fall prevention smartphone app. Cost coverage of a fall prevention app was classified as an attractive feature according to the Kano technique. Comparing measured WTP for fall risk prevention to Alzheimer prevention shows how small the amount of money is that potential patients would invest in a fall prevention service. WTP for an Alzheimer prevention was about $155 per month, whereas a median amount around €5 per month was revealed for a fall risk prevention [66]. Nevertheless, to the best of the authors’ knowledge, this is the first survey being able to price a fall prevention smartphone app and therefore is able to support developers in designing an app satisfying users’ expectations. Prior studies primarily investigated the clinical costs of fall patients as well as the amount of money saved by different intervention programs [67,68]. On the basis of the results of this survey, researchers, as well as practitioners, can better understand which product features are necessary to design a smartphone app that will be acceptable to potential users and also be cost-effective.

### Limitations

This study has several limitations related to its methodological design as well as the reported results. The open Web-based study was not representative because of regional recruiting in Germany via Clickworker. A bias in recruitment might lead to differences in the groups in the accessed fall risk or desired product features of a fall prevention app.

Furthermore, participants’ health status and quality of life were good within the sample. Therefore, results might differ with a sample of participants suffering from worse health status or who have poorer quality of life. Just a small portion had already experienced a fall incidence; therefore, rating of product features might change with a sample including individuals with a higher number of experienced fall incidents. Future research might address this topic by in-depth focus groups to design a fall prevention app, especially for already fallen older adults.

### Conclusions

Fall incidents are severe problems among the elderly [2]. A major problem in this context is that older adults are unaware of their potential fall risk as it rises slowly [4,5]. It is, therefore, necessary to offer older adults a low-threshold service to assess their own risk of falling. In view of the increasing use of health apps in society and especially in the group of elderly individuals, an app appears to be a useful long-term approach to helping older people to prevent falls [12-16].

The aim of this study was to determine potential product features of a fall prevention app adults aged older than 60 years would appreciate irrespective of whether they already experienced a fall or not.

In an exploratory approach, product features were ascertained that potential users would expect from a fall prevention smartphone app. To the best of the authors’ knowledge, this is the first study explicitly investigating this aspect. In total, 17 product features were investigated, which were derived from different recent research projects about fall prevention. Twelve aspects were determined to be “must-be” product features, including unobtrusive fall risk detection, decision making about necessary treatment, and offering physical exercises to reduce the risk of falling. Attractive features of a fall prevention app would include educational features such as a checklist for typical tripping hazards and a guide of action in case of a fall. Hence, the authors are of the opinion that such an app could be successfully adapted within a common app store. This may enable interested older adults to identify, monitor, and treat under the supervision of a health professional their risk of falls, albeit the effectiveness of such an app would need to be evaluated in follow-up research studies.

## Appendix

Multimedia Appendix 1 Introduction text of survey (German/English).

## Abbreviations

AFPS: Aachen Falls Prevention Scale
AFRIS: Attitudes Falls Related Intervention Scale
ANOVA: one-way analyses of variance
EQ5D-3L: EuroQol Questionnaire 3 Level
C: criteria
CS: customer satisfaction coefficient
EU: European Union
FES-I: short Falls Efficacy Scale-International
FOF: fear of falling
IQR: interquartile range
WTP: willingness to pay

## References

[1] Liu SW, Obermeyer Z, Chang Y, Shankar KN (2015). Frequency of ED revisits and death among older adults after a fall. Am J Emerg Med.

[2] Swift CG (2001). Care of older people: falls in late life and their consequences-implementing effective services. Br Med J.

[3] Klos K, Simons P, Mückley T, Karich B, Randt T, Knobe M (2017). [Fractures of the ankle joint in elderly patients]. Unfallchirurg.

[4] Toraman A, Yildirim NU (2010). The falling risk and physical fitness in older people. Arch Gerontol Geriatr.

[5] Russell K, Taing D, Roy J (2017). Measurement of fall prevention awareness and behaviours among older adults at home. Can J Aging.

[6] Knobe M, Siebert CH (2014). [Hip fractures in the elderly: Osteosynthesis versus joint replacement]. Orthopade.

[7] Pastor T, Gradl G, Klos K, Ganse B, Horst K, Andruszkow H, Hildebrand F, Pape H, Knobe M (2016). Displaced intra-articular calcaneal fractures: is there a consensus on treatment in Germany?. Int Orthop.

[8] Gradl G, Knobe M, Pape H, Neuhaus PV, Ring D, Guitton T (2015). Decision making in displaced fractures of the proximal humerus: fracture or surgeon based?. Int Orthop.

[9] Knobe M, Gradl G, Ladenburger A, Tarkin IS, Pape H (2013). Unstable intertrochanteric femur fractures: is there a consensus on definition and treatment in Germany?. Clin Orthop Relat Res.

[10] Pape H, Schemmann U, Foerster J, Knobe M (2015). The 'Aachen Falls Prevention Scale' - development of a tool for self-assessment of elderly patients at risk for ground level falls. Patient Saf Surg.

[11] Knobe M, Giesen M, Plate S, Gradl-Dietsch G, Buecking B, Eschbach D, van Laack W, Pape H (2016). The Aachen Mobility and Balance Index to measure physiological falls risk: a comparison with the Tinetti POMA Scale. Eur J Trauma Emerg Surg.

[12] Shen C, Wang MP, Chu JT, Wan A, Viswanath K, Chan SS, Lam TH (2017). Health app possession among smartphone or tablet owners in Hong Kong: population-based survey. JMIR Mhealth Uhealth.

[13] Krebs P, Duncan DT (2015). Health app use among US mobile phone owners: a national survey. JMIR Mhealth Uhealth.

[14] Mellone S, Tacconi C, Schwickert L, Klenk J, Becker C, Chiari L (2012). Smartphone-based solutions for fall detection and prevention: the FARSEEING approach. Z Gerontol Geriatr.

[15] Mertens A, Rasche P, Theis S, Bröhl C, Wille M (2017). Use of Information and Communication Technology in healthcare context by older adults in Germany: initial results of the Tech4Age longitudinal study. i-com.

[16] Rasche P, Wille M, Bröhl C, Theis S, Schäfer K, Knobe M, Mertens A (2018). Prevalence of health app use among older adults in Germany: national survey. JMIR Mhealth Uhealth.

[17] Seifert A, Schlomann A, Rietz C, Schelling HR (2017). The use of mobile devices for physical activity tracking in older adults’ everyday life. Digit Health.

[18] Higgins JP (2016). Smartphone applications for patients' health and fitness. Am J Med.

[19] Mendiola MF, Kalnicki M, Lindenauer S (2015). Valuable features in mobile health apps for patients and consumers: content analysis of apps and user ratings. JMIR Mhealth Uhealth.

[20] Bakker D, Kazantzis N, Rickwood D, Rickard N (2016). Mental health smartphone apps: review and evidence-based recommendations for future developments. JMIR Ment Health.

[21] Rasche P, Mertens A, Bröhl C, Theis S, Seinsch T, Wille M, Pape H, Knobe M (2017). The “Aachen fall prevention App” - a smartphone application app for the self-assessment of elderly patients at risk for ground level falls. Patient Saf Surg.

[22] Patrick K, Griswold WG, Raab F, Intille SS (2008). Health and the mobile phone. Am J Prev Med.

[23] Klasnja P, Pratt W (2012). Healthcare in the pocket: mapping the space of mobile-phone health interventions. J Biomed Inform.

[24] cele.coventry.

[25] Klenk J, Schwickert L, Palmerini L, Mellone S, Bourke A, Ihlen EA, Kerse N, Hauer K, Pijnappels M, Synofzik M, Srulijes K, Maetzler W, Helbostad JL, Zijlstra W, Aminian K, Todd C, Chiari L, Becker C, FARSEEING Consortium (2016). The FARSEEING real-world fall repository: a large-scale collaborative database to collect and share sensor signals from real-world falls. Eur Rev Aging Phys Act.

[26] Rimland JM, Abraha I, Dell'Aquila G, Cruz-Jentoft A, Soiza R, Gudmusson A, Petrovic M, O'Mahony D, Todd C, Cherubini A (2016). Effectiveness of non-pharmacological interventions to prevent falls in older people: a Systematic overview. The SENATOR project ONTOP series. PLoS One.

[27] profound.eu.

[28] Hayes N (2015). Fallcheck. Nurs Stand.

[29] Vaziri DD, Aal K, Gschwind YJ, Delbaere K, Weibert A, Annegarn J, de Rosario H, Wieching R, Randall D, Wulf V (2017). Analysis of effects and usage indicators for a ICT-based fall prevention system in community dwelling older adults. Int J Hum Comput Stud.

[30] Knobe M, Rasche P, Rentemeister L, Bliemel C, Bücking B, Bollheimer L, Pape HC (2018). [Evaluation of a simple screening tool for ambulant fall prevention]. Unfallchirurg.

[31] Best SJ, Krueger BS (2004). Internet data collection.

[32] Topolovec-Vranic J, Natarajan K (2016). The use of social media in recruitment for medical research studies: a scoping review. J Med Internet Res.

[33] Kano N, Seraku N, Takahashi F, Tsuji F (1984). Attractive quality and must-be quality. JSQC.

[34] Mikulić J, Prebežac D (2011). A critical review of techniques for classifying quality attributes in the Kano model. MSQ.

[35] Materla T, Cudney EA, Antony J (2017). The application of Kano model in the healthcare industry: a systematic literature review. Total Qual Manag Bus.

[36] Hejaili FF, Assad L, Shaheen FA, Moussa DH, Karkar A, AlRukhaimi M, Barhamein M, Al Suwida A, Al Alhejaili FF, Al Harbi AS, Al Homrany M, Attar B, Al-Sayyari AA (2009). Culture-related service expectations: a comparative study using the Kano model. Qual Manag Health Care.

[37] Mkpojiogu EO, Hashim NL (2016). Understanding the relationship between Kano model's customer satisfaction scores and self-stated requirements importance. Springerplus.

[38] Desteghe L, Kluts K, Vijgen J, Koopman P, Dilling-Boer D, Schurmans J, Dendale P, Heidbuchel H (2017). The health buddies app as a novel tool to improve adherence and knowledge in atrial fibrillation patients: a pilot study. JMIR Mhealth Uhealth.

[39] Santo K, Richtering SS, Chalmers J, Thiagalingam A, Chow CK, Redfern J (2016). Mobile phone apps to improve medication adherence: a systematic stepwise process to identify high-quality apps. JMIR Mhealth Uhealth.

[40] Sherrington C, Whitney JC, Lord SR, Herbert RD, Cumming RG, Close JCT (2008). Effective exercise for the prevention of falls: a systematic review and meta-analysis. J Am Geriatr Soc.

[41] Gillespie LD, Robertson MC, Gillespie WJ, Sherrington C, Gates S, Clemson LM, Lamb SE (2012). Interventions for preventing falls in older people living in the community. Cochrane Database Syst Rev.

[42] Shubert TE, Goto LS, Smith ML, Jiang L, Rudman H, Ory MG (2017). The Otago Exercise Program: innovative delivery models to maximize sustained outcomes for high risk, homebound older adults. Front Public Health.

[43] Thomas S, Mackintosh S, Halbert J (2010). Does the 'Otago exercise programme' reduce mortality and falls in older adults?: a systematic review and meta-analysis. Age Ageing.

[44] Schlomann A, von Storch K, Rasche P, Rietz C (2016). Means of motivation or of stress? The use of fitness trackers for self-monitoring by older adults. HBScience.

[45] Hajek A, Bock J, König H (2017). Which factors affect health care use among older Germans? Results of the German ageing survey. BMC Health Serv Res.

[46] Lee M, Newcomb J (1997). Applying the kano methodology to meet customer rquirements: Nasa's microgravity science program. QMJ.

[47] Fong D (1996). Using the self-stated importance questionnaire to interpret Kano questionnaire results. CQM.

[48] Berger C, Blauth R, Boger D, Bolster C, Burchill G, DuMouchel W, Pouliot F, Richter R, Rubinoff A, Shen D (1993). Kano's methods for understanding customer-defined quality. CQM.

[49] Brandl C, Rasche P, Bröhl C, Theis S, Wille M, Schlick C, Mertens A (2017). Incentives for the Acceptance of Mobility Equipment by Elderly People on the Basis of the Kano Model: A Human Factors Perspective for Initial Contact with Healthcare Products. Advances in Human Factors and Ergonomics in Healthcare.

[50] Arora S, ter Hofstede F, Mahajan V (2017). The implications of offering free versions for the performance of paid mobile apps. J Mark.

[51] Lin P, Cangelosi MJ, Lee DW, Neumann PJ (2013). Willingness to pay for diagnostic technologies: a review of the contingent valuation literature. Value Health.

[52] Sørensen K, Pelikan JM, Röthlin F, Ganahl K, Slonska Z, Doyle G, Fullam J, Kondilis B, Agrafiotis D, Uiters E, Falcon M, Mensing M, Tchamov K, van den Broucke S, Brand H, HLS-EU Consortium (2015). Health literacy in Europe: comparative results of the European health literacy survey (HLS-EU). Eur J Public Health.

[53] Röthlin F, Pelikan J, Ganahl K (2013). lbihpr.lbg.

[54] Agborsangaya C, Lahtinen M, Cooke T, Johnson J (2014). Comparing the EQ-5D 3L and 5L: measurement properties and association with chronic conditions and multimorbidity in the general population. Health Qual Life Outcomes.

[55] Panzer VP, Wakefield DB, Hall CB, Wolfson LI (2011). Mobility assessment: sensitivity and specificity of measurement sets in older adults. Arch Phys Med Rehabil.

[56] Kempen GI, Yardley L, van Haastregt JC, Zijlstra GA, Beyer N, Hauer K, Todd C (2008). The Short FES-I: a shortened version of the falls efficacy scale-international to assess fear of falling. Age Ageing.

[57] Ferrucci L, Guralnik JM, Studenski S, Fried LP, Cutler GB, Walston JD, Interventions on Frailty Working Group (2004). Designing randomized, controlled trials aimed at preventing or delaying functional decline and disability in frail, older persons: a consensus report. J Am Geriatr Soc.

[58] Auais M, Alvarado B, Guerra R, Curcio C, Freeman EE, Ylli A, Guralnik J, Deshpande N (2017). Fear of falling and its association with life-space mobility of older adults: a cross-sectional analysis using data from five international sites. Age Ageing.

[59] Neyer F, Felber J, Gebhardt C (2012). Entwicklung und Validierung einer Kurzskala zur Erfassung von Technikbereitschaft. Diagnostica.

[60] Yardley L, Donovan-Hall M, Francis K, Todd C (2006). Older people's views of advice about falls prevention: a qualitative study. Health Educ Res.

[61] Yardley L, Donovan-Hall M, Francis K, Todd C (2007). Attitudes and beliefs that predict older people's intention to undertake strength and balance training. J Gerontol B Psychol Sci Soc Sci.

[62] unipark.

[63] (2017). Clickworker.

[64] 42matters.

[65] Danbjørg DB, Villadsen A, Gill E, Rothmann MJ, Clemensen J (2018). Usage of an exercise app in the care for people with osteoarthritis: user-driven exploratory study. JMIR Mhealth Uhealth.

[66] Basu R (2013). Willingness-to-pay to prevent Alzheimer's disease: a contingent valuation approach. Int J Health Care Finance Econ.

[67] Irvine L, Conroy SP, Sach T, Gladman JR, Harwood RH, Kendrick D, Coupland C, Drummond A, Barton G, Masud T (2010). Cost-effectiveness of a day hospital falls prevention programme for screened community-dwelling older people at high risk of falls. Age Ageing.

[68] Jenkyn KB, Hoch JS, Speechley M (2012). How much are we willing to pay to prevent a fall? Cost-effectiveness of a multifactorial falls prevention program for community-dwelling older adults. Can J Aging.

