# Supplementary material for: Satisfying Product Features of a Fall Prevention Smartphone App and Potential Users’ Willingness to Pay: Web-Based Survey Among Older Adults
Source: JMIR Mhealth Uhealth. 2018 Mar 27;6(3):e75. doi: 10.2196/mhealth.9467 (PMC5893889; doi:10.2196/mhealth.9467)
Supplement: Multimedia Appendix 1 [file mhealth_v6i3e75_app1.pdf]

Introduction text of survey (German)

Liebe Teilnehmerin, Lieber Teilnehmer,

im Folgenden möchte ich Ihnen einige Fragen zu Ihren möglichen Wünschen und Anforderungen an eine App zur Sturzprävention stellen. Die Beantwortung dieser Fragen nimmt maximal 20 Minuten Ihrer Zeit in Anspruch.

Mit dem Klick auf weiter starten Sie in den Fragebogen.

Vielen Dank schon einmal vorab für Ihre Unterstützung!

Peter Rasche

Diese Umfrage wird im Rahmen eines Forschungsprojektes vom Bundesministerium für Bildung und Forschung durchgeführt (www.tech4age.de). Ihre Antworten werden im Sinne guter wissenschaftlicher Praxis anonym erhoben und dienen lediglich der wissenschaftlichen Forschung. Ihre Daten werden nicht an Firmen oder Dritte weitergegeben. Die Datenerhebung erfolgt im Einklang mit dem Verfahrensverzeichnis 10-15 beaufsichtigt durch den Datenschutzbeauftragten der RWTH Aachen University.'

Introduction text of survey (English translation)

Dear participant, dear participant,

In the following I would like to ask you some questions about your possible wishes and requirements for an app for fall prevention. Answering these questions takes a maximum of 20 minutes of your time.

Click on Continue to start the questionnaire.

Thank you very much in advance for your support!

Peter Rasche

This survey is conducted within the framework of a research project by the Federal Ministry of Education and Research (www.tech4age.de). The answers are anonymous in line with good scientific practice and are only used for scientific research. Your data will not be passed on to

companies or third parties. Data collection is carried out in accordance with the procedure list 10-15, supervised by the data protection officer at RWTH Aachen University.'
